# Comparative Pharmacokinetics Research of 13 Bioactive Components of Jieyu Pills in Control and Attention Deficit Hyperactivity Disorder Model Rats Based on UPLC-Orbitrap Fusion MS

**DOI:** 10.3390/molecules29061230

**Published:** 2024-03-10

**Authors:** Xuefang Liu, Yan Wan, Shuding Sun, Ting Wang, Ting Li, Qi Sun, Weiwei Zhang, Di Zhao, Yange Tian, Suxiang Feng

**Affiliations:** 1 Academy of Chinese Medical Sciences, Henan University of Chinese Medicine, Zhengzhou 450003, China; 2 Collaborative Innovation Center for Chinese Medicine and Respiratory Diseases Co-Constructed by Henan Province & Education Ministry of China, Zhengzhou 450046, China; 3 College of Pharmacy, Henan University of Chinese Medicine, Zhengzhou 450046, Chinaqisun137@163.com (Q.S.);; 4 Henan Taifeng Biology Science and Technology Co., Ltd., Kaifeng 475141, China

**Keywords:** depression, pharmacokinetic, UPLC-Orbitrap Fusion MS, Jieyu Pills (JYPs), attention deficit hyperactivity disorder (ADHD) model

## Abstract

Jieyu Pills (JYPs), a Chinese medicine consisting of 10 herbal elements, have displayed promising clinical effectiveness and low by-effects in the treatment of depression. Prior investigations mostly focused on elucidating the mechanism and therapeutic efficacy of JYPs. In our earlier study, we provided an analysis of the chemical composition, serum pharmacochemistry, and concentrations of the main bioactive chemicals found in JYPs. However, our precise understanding of the pharmacokinetics and metabolism remained vague. This study involved a comprehensive and meticulous examination of the pharmacokinetics of 13 bioactive compounds in JYPs. Using UPLC-Orbitrap Fusion MS, we analyzed the metabolic characteristics and established the pharmacokinetic parameters in both control rats and model rats with attention deficit hyperactivity disorder (ADHD) following oral administration of the drug. Before analysis, plasma samples that were collected at different time intervals after the administration underwent methanol pre-treatment with Puerarin used as the internal standard (IS) solution. Subsequently, the sample was chromatographed on a C18 column employing gradient elution. The mobile phase consisted of methanol solution containing 0.1% formic acid in water. The electrospray ionization source (ESI) was utilized for ionization, whereas the scanning mode employed was selected ion monitoring (SIM). The UPLC-Orbitrap Fusion MS method was subjected to a comprehensive validation process to assess its performance. The method demonstrated excellent linearity (r ≥ 0.9944), precise measurements (RSD < 8.78%), accurate results (RE: −7.88% to 8.98%), and appropriate extraction recoveries (87.83–102.23%). Additionally, the method exhibited minimal matrix effects (87.58–101.08%) and satisfactory stability (RSD: 1.52–12.42%). These results demonstrated adherence to the criteria for evaluating and determining biological material. The 13 bioactive compounds exhibited unique pharmacokinetic patterns in vivo. In control rats, all bioactive compounds except Ferulic acid exhibited linear pharmacokinetics within the dose ranges. In the ADHD model, the absorption rate and amount of most of the components were both observed to have increased. Essentially, this work is an important reference for examining the metabolism of JYPs and providing guidelines for clinical therapy.

## 1. Introduction

According to the World Health Organization, depression ranks as the third most prevalent cause of illness in the world [1,2,3]. By 2030, it is projected to surpass all other diseases and become the leading cause of morbidity. Roughly 66% of patients fail to heal and experience persistent depression, and we are all observing a consistent increasing pattern [3,4]. This has the potential to impact various aspects such as interpersonal relationships, societal interactions, educational institutions, and jobs. Over the last ten years, the frequency of depression has consistently risen. Several elements could contribute to the development of depression, including genetic components, social factors, health problems, and psychological factors [5]. Hence, antidepression medication has significant marketing potential. Despite generating billions of dollars in sales annually, a recent study revealed that the published literature exaggerated the effectiveness of several commonly prescribed antidepressants, such as fluoxetine, venlafaxine, nefazodone, paroxetine, milnacipran, and mianserin. The by-effects of several medications are unendurable. At the same time, drugs such as those used in Chinese Medicine (CM) have become increasingly popular and well-known in recent years due to the low risks associated with antidepressants [6,7]. Various experiments have investigated and verified the efficacy of CM in the treatment of depression. The active ingredients identified in antidepressants can generally be divided into saponins, flavonoids, alkaloids, polysaccharides, and others [8].

Recent studies have demonstrated that Jieyu Pills (JYPs) exhibit diverse pharmacological effects with minimal by-effects [9,10]. JYPs are utilized for the treatment of symptoms such as psychological depression, irritability, insomnia, and forgetfulness [11,12,13]. JYPs are a medicinal formula that originated from “The Essentials of the Golden Chamber” of the Han Dynasty and “Welfare Pharmacy” of the Song Dynasty. They consist of 10 herbs, namely, *Paeonia lactiflora* Pall., *Bupleurum falcatum* L., *Angelica sinensis* (Oliv.) Diels, *Curcuma aromatica* Salisb., *Smilax glabra* Roxb., *Lilium lancifolium* Thunb., *Morus alba* L., *Triticum aestivum* L., *Glycyrrhiza glabra* L., and *Ziziphus jujuba* Mill. (All the herbs’ names were obtained from MPNS (https://mpns.science.kew.org), accessed on 14 November 2023). Presently, the therapeutic effectiveness of JYPs in reducing anxiety, alleviating depression, and enhancing sleep quality has been well acknowledged [14].

**Figure 1:**
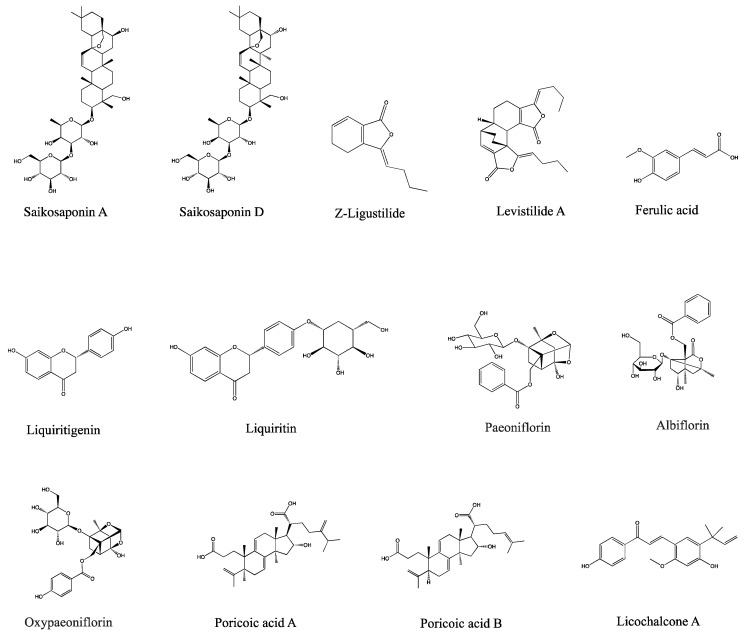
Chemical structures of 13 bioactive compounds: Saikosaponin A, Saikosaponin D, *Z*-Ligustilide, Ferulic acid, Liquiritigenin, Liquiritin, Paeoniflorin, Albiflorin, Oxypaeoniflorin, Poricoic acid A, Poricoic acid B, Levistilide A, and Licochalcone A.

Furthermore, our previous study successfully identified and detailed the components and molecular processes of JYPs, which contained a grand total of 188 components. A collection of 20 main bioactive compounds of JYPs was created to serve as a scientific basis for assessments and quality control of JYPs. However, animal experiments are required to have a comprehensive understanding of the pharmacokinetics and metabolic mechanisms of these bioactive compounds. Therefore, after analyzing the data from our initial experiment, we selected 13 major bioactive components of JYPs for pharmacokinetic research in both ADHD model and control rats, based on the results of drug-active compositions and serum chemistry studies. The components consist of Saikosaponin A, Saikosaponin D, *Z*-Ligustilide, Ferulic acid, Liquiritigenin, Liquiritin, Paeoniflorin, Albiflorin, Oxypaeoniflorin, Poricoic acid A, Poricoic acid B, Levistilide A, and Licochalcone A. Biological samples often have small quantities for sampling and generally contain low amounts of drugs. Biological samples also contain a multitude of natural compounds that could interrupt the determination results. Hence, the analytical techniques employed in pharmacokinetic research must satisfy precise requirements, with high selectivity and sensitivity. The UPLC-Orbitrap Fusion MS technology was therefore selected to analyze the specific pharmacokinetic pattern of 13 bioactive compounds in JYPs (Figure 1) in both control and ADHD rats.

## 2. Experimental Results

### 2.1. Method Validation

#### 2.1.1. Specificity Test

The specificity was assessed after comparing the chromatograms of plasma samples with added standard solutions (A), plasma samples taken after the injection of JYPs (B), and blank plasma samples (C). Figure S1 (Supplementary Materials) displays the outcomes of the specificity test. The results demonstrated that the instrumental response of all tested compounds exhibited a good level of sensitivity and distinctiveness. Furthermore, the endogenous components in the plasma and added components did not hinder the detection of the targeted compounds in the rats.

#### 2.1.2. Calibration Curve and Linear Range

Linear regression analysis was performed using the internal standard as the vertical coordinate to obtain the regression equation. Subsequently, the calibration curve was established based on the obtained results, as presented in Table 1. The results showed a good linear relationship with the concentration of the corresponding drugs in the linear concentration range (r ≥ 0.99), which met the needs of quantitative analysis.

#### 2.1.3. Precision and Accuracy

Rats were used to obtain plasma samples devoid of any substances. Subsequently, varying concentrations of control solutions (high, medium, and low) were introduced into the plasma samples, which were designated as quality control (QC) samples. Six samples were prepared and measured for three consecutive days with the same manipulation. The QC sample concentrations, as well as the precision and accuracy for both intra-day and inter-day measurements, were calculated. The findings indicated that the relative standard deviations (RSDs) for intra-day precision varied between 1.30% and 6.42%, whereas the RSDs for inter-day precision ranged within 1.46–8.78%. The relative errors (REs), which measured the accuracy within a single day and between different days, varied from −7.88 to 8.98% and from −7.82 to 6.66%, respectively. These values fell within the acceptable range for validating analytical procedures used on biological samples. The results are displayed in Table 2.

#### 2.1.4. Substrate Effect and Extraction Recoveries

The peak area of each compound was determined and recorded as A_std_ (A), A_is_ (A). The peak areas of the blank plasma samples with high, medium, and low concentrations of the mixed control solution, as detailed above, were recorded as A_std_ (B), A_is_ (B). The peak areas of each component and the internal standard were determined after treatment and were recorded as A_std_ (C), A_is_ (C). Six replicates of each sample were made in parallel. The formulae for the matrix effect and extraction recovery are as follows

$$Matrix\;effect\;(\%)=\frac{Astd\;(A)/Ais\;(A)}{Astd\;(B)/Ais\;(B)}\times 100$$

$$Extraction\;recovery\;(\%)=\frac{Astd\;(B)/Ais\;(B)}{Astd\;(C)/Ais\;(C)}\times 100$$

The matrix effect and extraction recovery of each component are shown in Table 3 and were within 87.58–101.08%, indicating no plasma matrix affecting each component, along with a good recovery rate (87.83–102.23%), in line with the requirements of the analytical methods for biological samples.

#### 2.1.5. Stability Test

QC samples of each concentration were taken to investigate the stability under three conditions: (1) short-term stability: placed at 4 °C for 12 h; (2) long-term stability: frozen at −80 °C for 30 days; and (3) freeze–thaw stability: kept at −80 °C for repeated freezing and thawing three times. In Table 4, the RSD ranges within 1.52–12.42%, with good stability.

Based on UPLC-MS technology, a multi-component analytical method was established and methodologically investigated for the analysis of JYPs in control rats. The endogenous components in plasma did not interfere with the components tested. The linear relationship between the components in the linear range was good (r ≥ 0.9939). The intra-day precision and inter-day precision RSD were less than 8.78%, the matrix effect ranged from 87.58% to 101.08%, and the recoveries were in the range of 87.83–102.23%, with good stability.

### 2.2. Pharmacokinetic Analysis of Control Group

**Figure 2:**
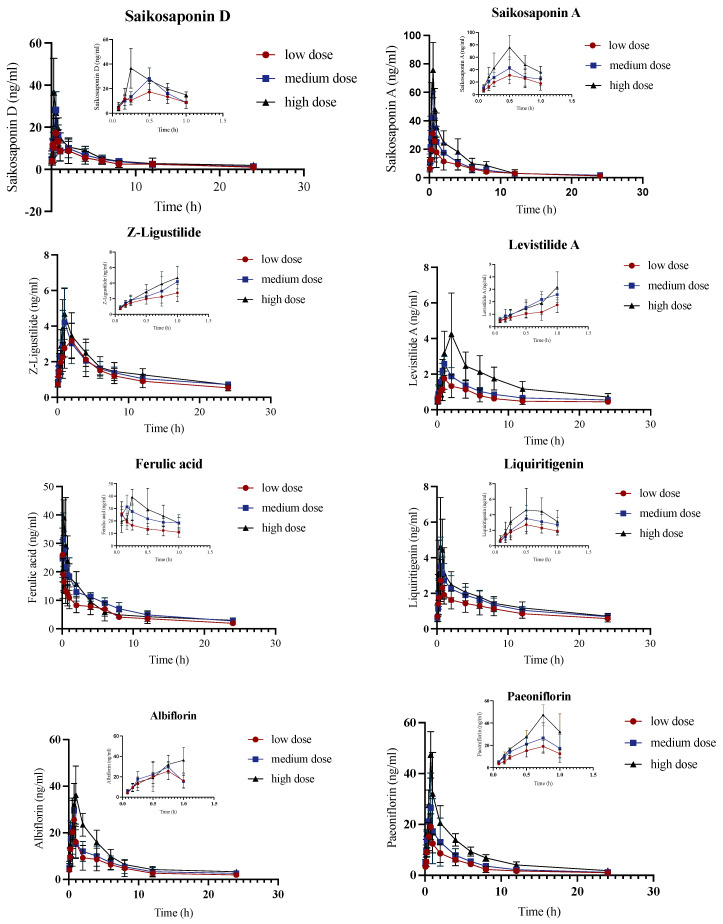

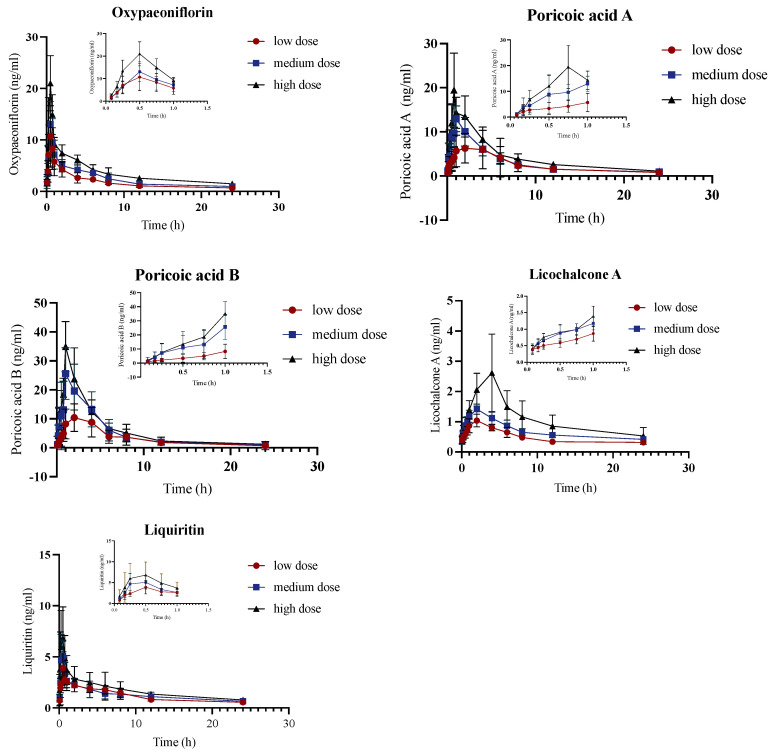
The concentration-time curves of the control model group were obtained by administering 13 bioactive chemicals through gavage to control rats (*n* = 6). The vertical bars represent standard deviations.

According to the established method for the analysis of serum biological samples, the pharmacokinetics of various components of rat serum were investigated after the administration of different dosages of JYPs to control rats. In Figure 2 and Table S1 (Supplementary Materials), the pharmacokinetic parameters of the bioactive components of different doses of the JYPs are varied. To assess whether the amount of exposure to 13 bioactive substances was directly related to the dosage, the linearity of ln(AUC_0-t_)–ln(dose) and ln(C_max_)–ln(dose) was analyzed. Hence, all bioactive substances, except for Ferulic acid, demonstrated linear pharmacokinetics (a positive correlation) in control rats at the given dosages. However, as the number of animals receiving each dose was restricted, additional verification was required. There was no association between the AUC_0-t_ of Ferulic acid and the increase in dosage. Nevertheless, the AUC values for Ferulic acid exhibited an increase at low and medium dosages but a decrease at high doses, as indicated in Table S1 (Supplementary Materials). This discovery suggests that Ferulic acid might demonstrate non-linear pharmacokinetic characteristics within the assessed dosages. Non-linear pharmacokinetics might arise due to several aspects associated with carrier-mediated absorption, first-pass effects, binding, excretion, or biotransformation [15]. In control rats, Saikosaponin A, Saikosaponin D, Ferulic acid, Liquiritigenin, Liquiritin, Paeoniflorin, and Oxypaeoniflorin were rapidly absorbed (T_max_ < 1 h). The contents of Saikosaponin A and Saikosaponin D were lower than those of Paeoniflorin and Oxypaeoniflorin in the JYPs. However, the characteristics of pharmacokinetics differed among the different groups, as the concentrations of Saikosaponin A, Saikosaponin D, Paeoniflorin, and Oxypaeoniflorin were not significantly different in control rats, suggesting that the gastrointestinal tract more readily absorbed Saikosaponin A and Saikosaponin D. The peak time was less than 0.75 h and the half-life was within 7.52–13.7 h, indicating that Saikosaponin A and Saikosaponin D could not only be absorbed rapidly by the gastrointestinal tract but also be eliminated in vivo, rarely generating residue and building up, indicating that Saikosaponin A and D might serve as potent components of JYPs. Poricoic acid A and Poricoic acid B were the major bioactive compounds exhibiting antidepression effects in *Smilax glabra* Roxb [16]. The peak time was less than 4 h and the half-life was within 7.61–12.23 h, which indicated that Poricoic acid A and Poricoic acid B were absorbed slowly in the gastrointestinal tract but metabolized quickly and were eliminated as soon as possible in vivo. Albiflorin and Paeoniflorin displayed comparable plasma concentration–time characteristics in ADHD rats. C_max_ was achieved in around 2 h, with approximately 80% removed within 12 h. Ferulic acid, Liquiritigenin, and Liquiritin’s peak time was less than 0.75 h, while t_1/2_ was within 9.53–16.81 h, indicating that these three compounds were absorbed quickly but metabolized slowly. Ferulic acid had a lower content in the JYPs but had a relatively high blood concentration. This suggested that the Ferulic acid was quickly absorbed in the gastrointestinal tract, leading to significant variations in the concentration of the medicine in the blood, which showed a significant level of bioavailability [17,18,19]. The peak time of *Z*-Ligustilide and Levistilide A was less than 2 h, and the half-life was within 14.36–23.99 h, which indicated that their absorption was slow. The clearance of *Z*-Ligustilide and Levistilide A was slow in vivo.

### 2.3. Pharmacokinetic Study of JYPs in ADHD Model Group

**Figure 3:**
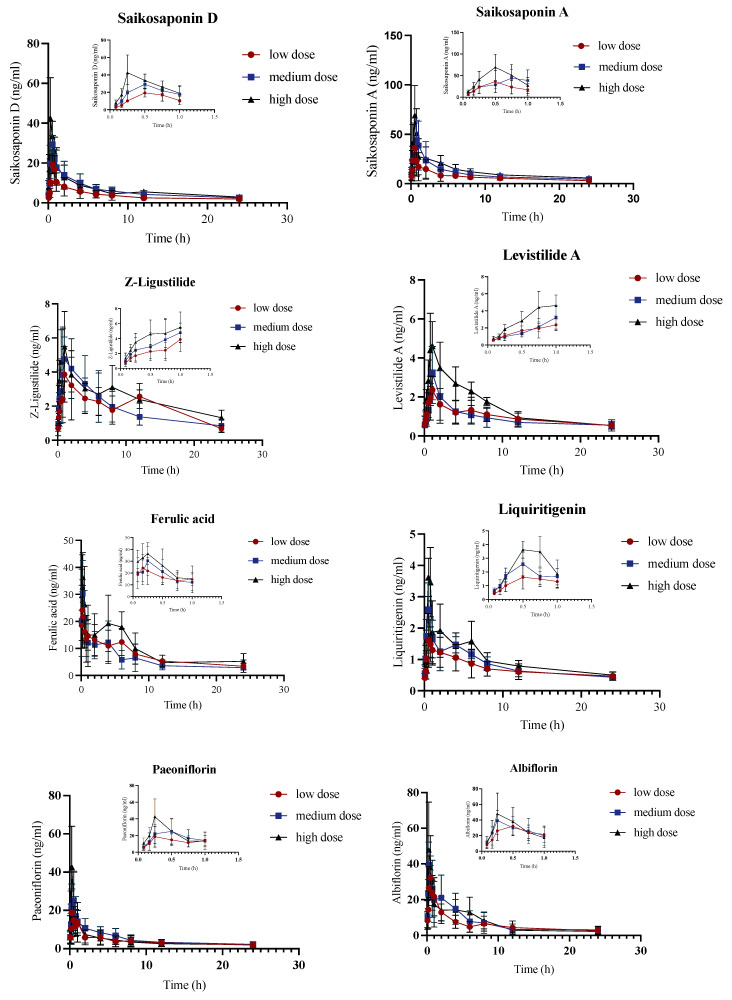

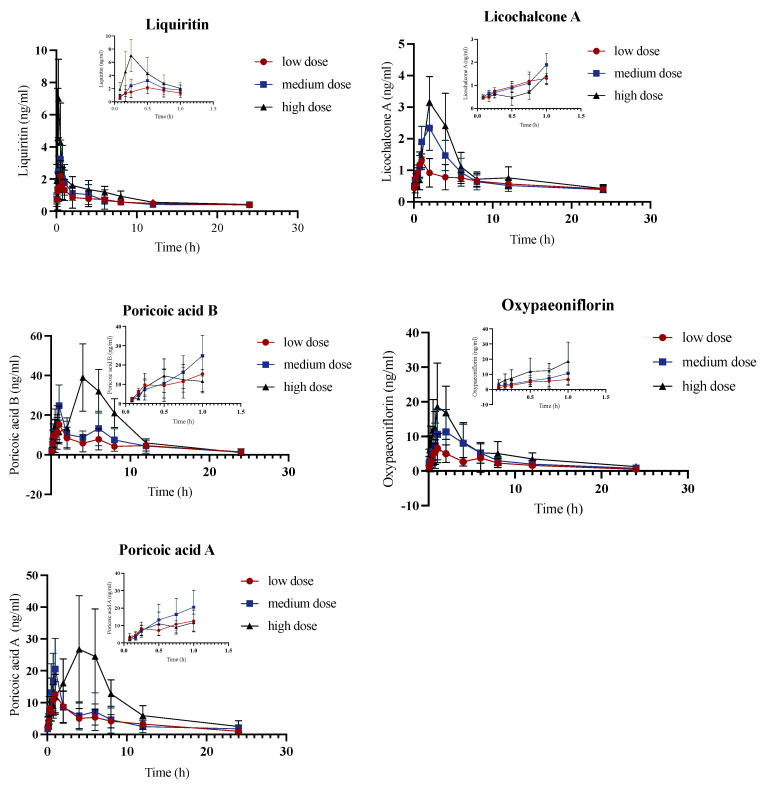
The concentration-time curves of the ADHD model group were obtained by administering 13 bioactive chemicals through gavage to ADHD model rats (*n* = 6).

Based on the established analytical method for plasma samples, a pharmacokinetic study was conducted on 13 constituents in the plasma of ADHD model rats after gavage of JYPs (Figure 3). The linearity of ln(AUC_0-t_)–ln(dose) and ln(C_max_)–ln(dose) was analyzed in the same way. Hence, all bioactive substances, except for *Z*-Ligustilide, Ferulic acid, Paeoniflorin, and Albiflorin, demonstrated linear pharmacokinetics (a positive correlation) in ADHD rats. According to the pharmacokinetic parameters, it was observed that Saikosaponin A, Saikosaponin D, *Z*-Ligustilide, Ferulic acid, Albiflorin, and Poricoic acid A in ADHD model rats followed the linear pharmacokinetic process (Table S2, Supplementary Materials). The results showed that Ferulic acid, Poricoic acid A, and Poricoic acid B were lower in the JYPs but with a high C_max_, which means that they were easily absorbed by the gastrointestinal tract. Six components (Saikosaponin A, Saikosaponin D, Liquiritigenin, Liquiritin, Paeoniflorin, and Albiflorin) could be rapidly absorbed in the gastrointestinal tract, with peak times of less than 1 h. The C_max_ values of Poricoic acid A and B in the ADHD rats were high, with peak times of less than 4 h and t_1/2_ of less than 10.32 h, suggesting that Poricoic acid A and B were absorbed slowly and were eliminated in a short time, with no residue or accumulation in ADHD rats.

### 2.4. Pharmacokinetic Comparison of JYPs in Control Rats and ADHD Model Rats

**Figure 4:**
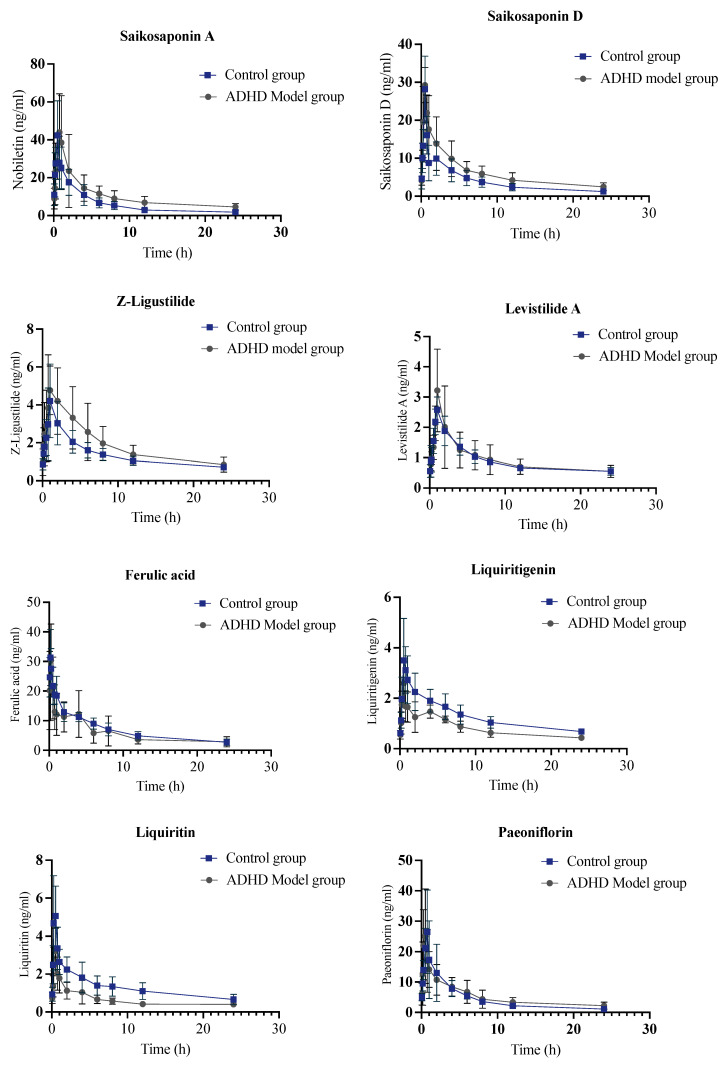

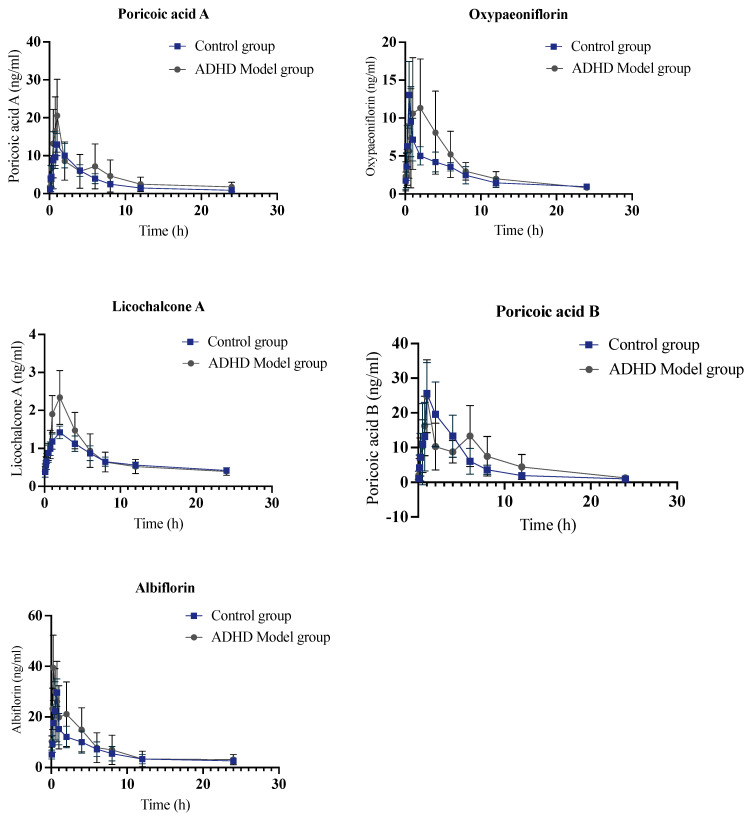
Medium-dose plasma concentration-time curves for the control group and ADHD model group were obtained after administering JYPs to control rats via gavage (*n* = 6).

All the results obtained and shown in Figure 4 (using the medium dose as an example) illustrate the comparison between the control group and the ADHD group. The results revealed that the AUC_(0-t)_ of Saikosaponin A was significantly greater in the model rats of the high-dose administration group. The total exposure to Saikosaponin A in the high-dose model rats was higher than that in the control rats. The MRT_(0-t)_ of Saikosaponin A was significantly higher in the ADHD rats at all concentrations. This indicated that the mean residence time of Saikosaponin A was significantly longer in the ADHD rats than in the control rats receiving the same dose. Therefore, it could be inferred that the duration of Saikosaponin A exposure in the model rats was extended. Previous studies have revealed that Saikosaponin A might exert antidepressant effects by upregulating the expression level of PRRT2 in the hippocampus [19,20]. The exposure to Saikosaponin D in the medium- and high-dose ADHD model rats was greater because of a higher AUC. Furthermore, Saikosaponin D stayed within the high-dose model rats for a longer duration in vivo with a higher MRT value. Research has shown that Saikosaponin D could ameliorate depression behaviors in rats by downregulating NF-κB and miR-155 and upregulating FGF_2_ [21,22]. Above all, Saikosaponin A and Saikosaponin D were postulated to be the pivotal constituents of relief medications used for the management of depression. The AUC_(0-t)_ of Liquiritigenin was significantly lower in the ADHD rats at all dosages. Additionally, the concentration of Liquiritigenin was significantly higher in the model rats in the medium- and high-dose groups. There was a decrease in the uptake of Liquiritigenin and an increase in metabolizing efficiency with the pathological condition. There is evidence that Liquiritin might undergo biotransformation in the rat gut to derive Isoliquiritigenin and Liquiritigenin [21]. Liquiritin is absorbed through passive diffusion and follows first-order kinetics, so its bioavailability is limited [22]. However, Liquiritin could serve as a precursor for Liquiritigenin and possesses greater pharmacological potency. Hence, the breakdown of Liquiritin could be ascribed to a rise in the plasma levels of Isoliquiritigenin and Liquiritigenin throughout absorption [23]. The ADHD rats showed a significantly higher T_max_ value for Poricoic acid A. The absorption efficiency of Poricoic acid A was increased in the ADHD group, and its peak time was shorter. Additionally, the ADHD rats exhibited a significantly higher C_max_. Thus, the total exposure to Poricoic acid A in the model rats in the low- and medium-dose groups was higher. The metabolism of Poricoic acid B was faster in the pathological condition as a result of a shorter t_1/2_.

The T_max_ of Albiflorin in the model rats in the high-dose group was significantly shorter. The ADHD rats absorbed Albiflorin more quickly at a high dose and reached the peak concentration faster. The C_max_ of Albiflorin in the model rats in the middle- and high-dose administration groups was significantly greater than that of Albiflorin in the control rats. This suggests that the overall exposure to Albiflorin in the middle- and high-dose model rats was higher. The uptake of Albiflorin increased in the rats under the pathological condition, and Albiflorin is hypothesized to play a key role in the treatment of depression. Albiflorin and Paeoniflorin are isomers. Several studies have been undertaken to examine their efficacy in treating depression [24]. Studies have also indicated that the therapeutic effects of Albiflorin are closely linked to the quick correction of a range of typical metabolic abnormalities in the hippocampus [25]. The evidence for this is that Albiflorin effectively suppresses the excessive production of cytosolic phospholipases A_2_, thus rectifying the abnormality in the kynurenine route of tryptophan metabolism and promoting increased production of serotonin in the hippocampus [26,27,28].

Studies are scarce on the use of Levistilide in the field of depression. Nevertheless, it has been inferred that Levistilide possesses an anti-oxidative impact. Levistilide inhibits neuronal cell death, improves the functioning of the nicotinic system, and reduces neuroinflammation in living organisms [29]. According to clinical research, stress triggers the activation of microglia, leading to a rise in the production of pro-inflammatory cytokines. However, it has been found that Ferulic acid could block the excitation of microglia and reduce the levels of pro-inflammatory cytokines [30,31,32,33]. Moreover, Z-ligustilide exhibits substantial potential in the areas of antidepression and the regulation of gut flora, among other aspects [34]. However, the oral bioavailability of Z-ligustilide in rats was relatively poor following oral dosing. The liver’s considerable first-pass metabolism could be one of the causes of this phenomenon. Z-ligustilide was seen to be readily absorbed. The rate at which Z-ligustilide spread from the blood to the tissues was moderate, but the rate at which it was eliminated varied significantly. The observed variance can be ascribed to the synergistic effect of Z-ligustilide and serum protein, perhaps leading to drug retention. It is worth mentioning that Z-ligustilide is not well absorbed when taken orally due to its limited bioavailability [35]. Above all, following the administration of JYPs via gavage in ADHD model rats, several components exhibited distinct characteristics in terms of their absorption rate, total drug absorption, and drug metabolism rate when compared to control rats.

## 3. Materials and Methods

### 3.1. Chemical and Reagents

Saikosaponin A (Batch No. MUST-22080810, purity: 99.74%), Saikosaponin D (Batch No. MUST-22021812, purity: 98.73%), Oxypaeoniflorin (Batch No. MUST-22050801, purity: 99.46%), Albiflorin (Batch no. MUST-22112310, purity: 99.96%), Poricoic acid A (Batch No. MUST-22120101, purity: 98.21%), Poricoic acid B (Batch No: MUST-22120102, purity: 98.04%), *Z*-Ligustilide (Batch No. MUST-22102401, purity: 99.72%), Licochalcone A (Batch No. MUST-17032501, purity: 99.92%), Levistilide A (Batch No.: MUST-22110119, purity ≥ 98.31%), and Liquiritigenin (Batch No.: MUST-17022104, purity ≥ 99.07%) were purchased from Chengdu Must Biotechnology Co., Ltd, Chengdu, China. Paeoniflorin (Batch No. 110736-202145, purity: 94.6%), Liquiritin (Batch No. 111610-201908, purity: 95.0%), and Ferulic acid (Batch No. 110773-201915, purity: 99.4%) were purchased from China Academy of Food and Drug Control, Beijing, China. JYPs (4 g/batch) were supplied by Henan Taifeng Pharmaceutical Company, Kaifeng, China, Batch No. 09220507. Acetonitrile (Lot No. F21M81203) was purchased from Thermo Fisher, MA, USA. Methanol (TEDIA, Fairfield, USA), formic acid (Thermo Fisher Scientific, Waltham, MA, USA), and other reagents were analytically pure.

### 3.2. Instruments

The UPLC-Orbitrap Fusion Mass Spectrometer, Heraeus Multifuge X_1_R Refrigerated Benchtop High Speed Centrifuge, Xcalibur workstation, 933-type ultra-low-temperature refrigerator and ME2020 high-speed centrifuge (Thermo Fisher Scientific, Waltham, MA, USA); Milli-QPOD ultrapure water meter (Merck, Darmstadt, Germany), ME204E one-hundred-thousandth analytical balance (Mettler-Toledo Shanghai Instrument Co., Ltd., Shanghai, China), KQ-500B ultrasonic cleaner (Ultrasonic Instrument Co. Scientific, Kunshan, China), XH-C-type oscillator (Acepom Instrument Manufacturing Co., Ltd., Jintan, China), and BE-3100-type Super Mixer (Chirinbeier Instrument Manufacturing Co., Ltd., Haimen, China) were used in this study. The data were computed by non-compartmental analysis, utilizing Kinetica 5.1 (Innaphase, Waltham, MA, USA) software for pharmacokinetic analysis. Prism 9 Plotting software was used to generate the pharmacotemporal curves. The statistical software SPSS 19.0 (IBM, Armonk, NY, USA) was employed for the analysis of all the parameters.

### 3.3. Experimental Animals

For the control experiment, healthy male Sprague Dawley (SD) rats of SPF grade (240 ± 20 g) were purchased from Sipeifu (Beijing) Biotechnology Co., Ltd. (Beijing, China), Animal Quality Certificate No. 110324220102348616. For the ADHD model rats, healthy male SHR rats of SPF grade, weighing 220 ± 20 g, were purchased from SBF (Beijing) Biotechnology Co., Ltd. (Beijing, China), License No. SCXK (Yu) 2019-0010. The animals were accommodated in the Animal Center of Henan University of Traditional Chinese Medicine, which successfully underwent an ethical audit with ethics number DWLL202208003. The animals were bred under controlled conditions at a temperature of 24 °C and a relative humidity of 50 ± 2%. Before the experiment, the animals abstained from eating for 12 h and had unrestricted access to water. The feeding and experimental research conducted on the experimental animals adhered to the laws outlined in the Management of Experimental Animals in Henan Province.

### 3.4. Preparation of JYP Gavage Solution

JYPs were ground into a fine powder and sieved through 60-mesh sieves; then, 20 g of fine JYP powder was taken, having been weighed precisely. A precise volume of 50 mL of pure water was added and mixed well to prepare a 0.4 g/mL suspension solution of JYPs, which was then stored in the refrigerator at 4 °C.

### 3.5. Preparation of Control Solution

Ferulic acid (11.12 mg), Poricoic acid A (10.92 mg), Poricoic acid B (11.71 mg), Saikosaponin A (13.42 mg), Saikosaponin D (12.10 mg), Albiflorin (9.42 mg), Oxypaeoniflorin (11.15 mg), Paeoniflorin (9.84 mg), *Z*-Ligustilide (10.12 mg), Levistilide A (11.62 mg), Liquiritigenin (11.21 mg), Liquiritin (10.40 mg), and Licochalcone A (10.80 mg) were precisely weighed and placed in a 10 mL volumetric flask; methanol was added under ultrasonication to dissolve the compounds, which were then diluted to scale under shaking and refrigerated at 4 °C on standby as control reserve solutions. Puerarin (11.60 mg) was weighed precisely and placed in a 10 mL flask. Methanol was added to dissolve the compound, which was then diluted to scale as an internal standard stock solution and refrigerated at 4 °C on standby.

In this experiment, the components Baicalein, Nobiletin, Myricetin, and Puerarin were investigated. The internal standard chosen was Puerarin, which had the benefits of a consistent and robust detection signal, as well as perfect separation from the compounds being analyzed.

### 3.6. Drug Administration 

This study involved 24 healthy male SD rats with a body mass of 220 ± 20 g and 24 healthy male ADHD rats with a body mass of 220 ± 20 g. Subjects abstained from food for a duration of 12 h before the experiment and consumed water without restriction. Both kinds of rats were randomly divided into four groups: a blank group, given an equal dose of water intragastrically, and 3 different administration groups: rats given the equivalent human dose of JYPs by gavage in doses of 1.080 g/kg/d, 2.160 g/kg/d, and 4.320 g/kg/d. The dosage equivalent to the amount of a single dose administered for each dose is shown in Table 5. Tail vein blood was collected at 0.083, 0.25, 0.5, 0.75, 1, 1.5, 2, 3, 4, 6, 8, 12, 18, and 24 h after dosing. About 0.5 mL of blood was collected in sodium heparin centrifugal tubes, then centrifuged at 4 °C for 10 min at a speed of 3500r/min. We took the upper layer and placed it at −80 °C for preservation.

### 3.7. Pretreatment of Biological Samples 

We took 200 μL of plasma, added 600 μL of methanol solution and 50 μL of internal standard (100 ng/mL), and subjected it to shaking for 5 min, followed by centrifugation at 4 °C at 12,000 r/min for 10 min. The supernatant was collected and allowed to dry, then reconstituted with 50 μL of 50% methanol and centrifuged at 4 °C at 14,000 r/min for a further 10 min. The supernatant after this process was taken as the test solution. A volume of 5 μL was used for UPLC-MS analysis. The drug concentration in plasma was calculated with the aid of the internal standard.

### 3.8. Chromatography–Mass Spectrometry Detection Conditions

Chromatographic conditions: Hypersil GOLD (100 × 2.1 mm, 1.9 μm) column; column temperature 35 °C; mobile phase acetonitrile (A)/0.1% formic acid aqueous solution (B); gradient elution; gradient elution program: 0–0.5 min (95% B–95% B), 0.5–8 min (95% B–50% B), 8–14 min (50% B–20% B), 14–20 min (20% B–5% B), 20–22 min (5% B–5% B), 22–25 min (95% B–95% B); flow rate 0.2 mL/min; injection volume 5 μL. The ion source was electrospray ionization (ESI), with positive and negative ion mode spray voltages of 3.50 and 3.0 kV, an ion transfer tube temperature of 300 °C, nitrogen as the carrier gas, a sheath gas pressure of 35 arb, an auxiliary gas pressure of 8 arb, and an auxiliary heater temperature of 275 °C. The scanning mode was multi-reaction ion detection, and the detected ions are shown in Table 6.

### 3.9. Method Validation

The method validation successfully demonstrated linearity, specificity, sensitivity, precision, accuracy, recovery, matrix effect, and stability. The analysis of blank plasma samples allowed for the identification of the specificity for potential interference by substances and internal standards. The calibration curves for the quantitative evaluation were determined by graphing the peak area ratio (y) of each compound to IS versus the nominal concentration (x) by using (1/x^2^) least-squares linear regression. The precision and accuracy were assessed by analyzing 6 parallel QC samples over 3 days. The relative error (RE) and relative standard deviation (RSD) were employed to illustrate the variations in precision and accuracy within a single day and across multiple days. The stability assessment of QC samples in blank plasma was conducted at four distinct concentrations in three different storage conditions. These conditions included storage at 4 °C for 12 h, freezing at −80 °C for 30 days, and subjecting samples to three freeze–thaw cycles from −20 °C to room temperature. The recovery was evaluated by comparing the peak areas of the analyte standards obtained from extracted samples with those of post-extracted samples that were artificially spiked with the analytes. The matrix effects were assessed by comparing the spiked post-extracted blank plasma samples with the corresponding standard solutions.

### 3.10. Statistical Analyses and Data Processing

The pharmacokinetics of JYPs in rats were investigated using the proven Orbitrap Fusion MS technology to examine the dynamic variations in the active components in rats. The pharmacokinetic parameters of all elements in each dosage group were estimated by utilizing Kinetica 5.1 software (Innaphase, Waltham, MA, USA) for pharmacokinetic analyses. Plotting software was used to generate the pharmacotemporal curves. The software SPSS 19.0, developed by IBM in Armonk, NY, USA, was utilized to analyze all the parameters. The data were plotted in graphs of drug–time curves by GraphPad Prism 9 plotting software. Independent sample tests were performed after natural logarithmic transformation for AUC_(0-t)_ and C_max_. The non-parametric Mann–Whitney test was applied for T_max_, t_1/2_, and MRT_(0–t)_ (*p* < 0.05 was considered statistically significant).

## 4. Conclusions

To summarize, a dependable, swift, and consistent Orbitrap Fusion MS technique was developed to quantify the levels of 13 main bioactive constituents of JYPs in both ADHD model and control rats. Distinct pharmacokinetic characteristics of the 13 bioactive substances were discovered. Above all, this study serves as an essential resource for investigating the process of JYP absorption and offers valuable guidance for clinical medicine.

## Figures and Tables

**Table 1:** Regression equations, linear ranges, and correlation coefficients.

Compound	Calibration Curve	Linear Range (ng/mL)	r
Saikosaponin A	y = 0.0039x + 0.0527	0.53–334.12	0.9944
Saikosaponin D	y = 0.0039x + 0.0172	0.47–298.65	0.9979
*Z*-Ligustilide	y = 0.0164x + 0.3872	0.39–99.72	0.9977
Levistilide A	y = 0.0249x + 0.5986	0.26–133.65	0.9954
Ferulic acid	y = 0.0017x + 0.0083	0.44–275.83	0.9993
Liquiritigenin	y = 0.0265x + 0.1190	0.22–109.96	0.9992
Liquiritin	y = 0.018x + 0.0918	0.39–99.8	0.9993
Paeoniflorin	y = 0.0001x + 0.0038	0.37–231.77	0.9944
Albiflorin	y = 0.0003x − 0.0012	0.37–236.9	0.9980
Oxypaeoniflorin	y = 0.0012x + 0.0076	0.44–110.4	0.9986
Poricoic acid A	y = 0.0105x + 0.2400	0.42–107.04	0.9959
Poricoic acid B	y = 0.0045x + 0.0166	0.45–114.7	0.9986
Licochalcone A	y = 0.1271x + 0.2547	0.21–107.91	0.9987

**Table 2:** Precision and accuracy of components in plasma ($\bar{X}$ ± SD, *n* = 6).

Compound	Concentration	Intra-Day			Inter-Day		
		Mean ± SD	Accuracy	Precision	Mean ± SD	Accuracy	Precision
	ng/mL	ng/mL	RE, %	RSD, %	ng/mL	RE, %	RSD, %
Saikosaponin A	334.12	338.83 ± 10.69	1.41	3.16	336.59 ± 25.59	6.66	1.46
	167.06	167.65 ± 5.61	0.35	3.35	163.66 ± 4.12	1.42	4.16
	0.53	0.50 ± 0.02	−6.17	3.58	0.52 ± 0.03	−2.64	6.19
Saikosaponin D	298.65	286.23 ± 7.83	−4.16	2.74	283.88 ± 11.11	−4.94	3.91
	149.32	136.60 ± 6.17	0.32	4.52	143.01 ± 7.40	0.32	5.17
	0.48	0.42 ± 0.02	1.71	4.82	0.44 ± 0.03	1.71	6.68
*Z*-Ligustilide	99.72	103.01 ± 4.68	3.29	4.54	99.43 ± 4.54	3.30	2.46
	49.86	50.42 ± 1.64	1.13	3.25	45.16 ± 3.20	0.83	2.58
	0.20	0.20 ± 0.01	−2.22	5.45	0.19 ± 0.01	−4.13	3.27
Levistilide A	133.65	137.57 ± 4.37	2.93	3.18	133.81 ± 5.30	0.12	3.96
	66.82	64.34 ± 2.92	−3.72	4.54	61.59 ± 3.90	−7.82	6.32
	0.26	0.24 ± 0.01	−7.88	3.87	0.24 ± 0.02	−4.43	5.88
Ferulic acid	275.83	280.29 ± 5.39	1.62	1.92	270.57 ± 16.60	−1.91	6.14
	137.92	135.87 ± 7.97	−1.49	5.86	135.87 ± 7.97	−5.61	8.78
	0.44	0.43 ± 0.02	−2.96	4.65	0.41 ± 0.06	−3.93	5.65
Liquiritigenin	109.96	111.12 ± 2.31	1.05	2.08	106.17 ± 4.74	−2.73	4.67
	54.98	54.13 ± 2.46	−1.54	4.54	52.07 ± 4.78	−1.87	4.31
	0.22	0.21 ± 0.00	−6.13	1.30	0.21 ± 0.02	−4.39	5.98
Liquiritin	99.80	104.48 ± 1.90	4.69	1.82	99.16 ± 3.72	−0.64	3.75
	49.90	48.44 ± 3.11	−2.92	6.42	46.29 ± 1.78	−7.24	3.84
	0.39	0.36 ± 0.01	−7.90	3.24	0.37 ± 0.02	−4.45	5.63
Paeoniflorin	237.77	230.19 ± 13.02	−3.19	5.66	236.63 ± 16.46	−0.48	6.96
	115.89	106.94 ± 4.70	−7.72	4.39	102.51 ± 8.40	6.05	2.40
	0.37	0.40 ± 0.01	8.98	3.70	0.35 ± 0.02	−0.68	4.73
Albiflorin	236.90	230.32 ± 8.11	−2.78	3.52	236.71 ± 11.63	−0.08	4.91
	118.45	122.04 ± 2.41	3.03	1.98	105.95 ± 6.58	1.56	3.60
	0.37	0.36 ± 0.02	−2.48	4.44	0.35 ± 0.02	−4.98	4.65
Oxypaeoniflorin	110.40	109.05 ± 1.12	−1.22	1.03	106.90 ± 5.92	2.95	2.42
	55.20	53.77 ± 2.82	−2.59	5.25	48.93 ± 2.48	−0.71	3.55
	0.44	0.42 ± 0.01	−4.23	2.52	0.44 ± 0.02	−0.68	4.17
Poricoic acid A	107.04	104.64 ± 5.37	−2.25	5.14	105.15 ± 2.17	−1.77	2.07
	53.52	56.00 ± 2.52	4.63	4.50	47.39 ± 2.61	−0.96	4.86
	0.42	0.41 ± 0.01	−2.79	2.81	0.42 ± 0.02	0.88	5.87
Poricoic acid B	114.70	113.56 ± 2.46	−0.99	2.17	104.84 ± 4.25	−0.32	2.48
	57.35	56.44 ± 1.24	−1.59	2.20	48.90 ± 2.67	−0.98	3.05
	0.45	0.45 ± 0.02	−0.40	4.41	0.45 ± 0.02	0.26	4.61
Licochalcone A	107.91	104.80 ± 3.22	−2.88	3.08	103.65 ± 2.58	−0.23	2.33
	53.96	53.95 ± 2.21	1.06	3.53	48.21 ± 2.24	1.21	5.36
	0.21	0.20 ± 0.01	−3.30	2.71	0.20 ± 0.01	1.61	3.63

**Table 3:** Extraction recovery and matrix effects ($\bar{X}$ ± SD, *n* = 6).

Compound	Concentration Added	Matrix Effect		Extraction Recovery	
	(ng/mL)	Mean ± SD (%)	RSD (%)	Mean ± SD (%)	RSD (%)
Saikosaponin A	334.12	92.43 ± 3.31	3.59	90.12 ± 2.48	2.75
	167.06	94.11 ± 5.35	5.69	92.17 ± 4.31	4.68
	0.53	90.93 ± 6.05	6.65	88.33 ± 6.94	7.86
Saikosaponin D	298.65	95.28 ± 4.82	5.06	91.15 ± 6.00	6.58
	149.32	92.86 ± 5.38	5.79	88.44 ± 4.48	5.07
	0.48	96.43 ± 7.63	7.91	90.67 ± 6.57	7.25
*Z*-Ligustilide	99.72	91.10 ± 5.95	6.53	90.07 ± 3.35	3.72
	49.86	97.15 ± 4.82	4.96	92.93 ± 4.39	4.73
	0.20	87.78 ± 6.41	7.30	93.24 ± 3.07	3.30
Levistilide A	133.65	88.60 ± 3.82	4.31	88.52 ± 3.73	4.22
	66.82	97.45 ± 4.89	5.02	90.58 ± 7.33	8.10
	0.26	94.08 ± 8.04	8.55	92.43 ± 5.81	6.29
Ferulic acid	275.83	95.58 ± 5.90	6.18	94.51 ± 2.97	3.14
	137.92	92.03 ± 7.10	7.72	91.20 ± 3.98	4.36
	0.44	90.01 ± 6.50	7.22	89.99 ± 4.23	4.70
Liquiritigenin	99.80	94.15 ± 7.07	7.51	94.04 ± 3.48	3.70
	49.90	87.59 ± 4.98	5.69	87.83 ± 7.88	8.97
	0.39	96.11 ± 4.96	4.88	90.97 ± 4.30	4.73
Liquiritin	109.96	91.46 ± 3.43	3.75	88.13 ± 7.37	8.37
	54.98	101.24 ± 4.44	4.39	92.31 ± 4.86	5.27
	0.22	98.45 ± 6.07	6.17	90.99 ± 3.16	3.47
Paeoniflorin	231.77	93.78 ± 5.31	5.66	93.15 ± 6.20	6.65
	115.89	94.40 ± 6.66	7.06	89.84 ± 5.46	6.08
	0.37	91.78 ± 6.73	7.34	102.23 ± 8.76	8.57
Albiflorin	236.90	93.91 ± 5.66	6.02	91.87 ± 4.18	4.54
	118.45	92.65 ± 3.65	3.93	88.66 ± 4.16	4.70
	0.37	89.92 ± 5.60	6.23	93.31 ± 3.22	3.45
Oxypaeoniflorin	110.40	94.84 ± 4.28	4.51	93.85 ± 2.92	3.12
	55.20	93.51 ± 7.92	8.47	89.35 ± 5.93	6.64
	0.44	89.59 ± 5.92	6.61	90.80 ± 7.28	8.01
Poricoic acid A	107.04	100.13 ± 3.67	3.67	94.65 ± 3.58	3.78
	53.52	91.97 ± 4.20	4.57	86.48 ± 4.09	4.73
	0.42	89.52 ± 4.07	4.55	92.88 ± 4.48	4.82
Poricoic acid B	114.70	88.84 ± 5.55	6.18	87.96 ± 4.64	5.27
	57.35	100.83 ± 2.96	2.94	89.46 ± 4.66	5.21
	0.45	94.04 ± 3.90	4.15	90.74 ± 5.53	6.10
Licochalcone A	107.91	94.24 ± 5.22	5.54	93.04 ± 5.80	6.23
	53.96	99.05 ± 5.16	5.21	94.65 ± 1.15	1.22
	0.21	97.01 ± 4.95	5.10	90.59 ± 6.99	7.71

**Table 4:** Stability test results ($\bar{X}$ ± SD, *n* = 6).

Compound	Concentration	Short-Term Stability		Long-Term Stability		Freeze–Thaw Stability	
		Mean ± SD	RSD	Mean ± SD	RSD	Mean ± SD	RSD
	ng/mL	ng/mL	%	ng/mL	%	ng/mL	%
Saikosaponin A	334.12	333.79 ± 5.23	1.57	333.87 ± 8.64	2.59	329.84 ± 6.72	2.04
	167.06	163.03 ± 6.85	4.20	157.57 ± 7.50	4.76	162.70 ± 2.48	1.52
	0.53	0.52 ± 0.02	4.10	0.53 ± 0.02	4.42	0.54 ± 0.02	3.10
Saikosaponin D	298.65	292.71 ± 9.07	3.10	286.73 ± 7.86	2.74	297.24 ± 6.14	2.06
	149.32	149.80 ± 6.27	4.19	144.97 ± 6.67	4.60	148.89 ± 2.57	1.72
	0.48	0.49 ± 0.03	6.27	0.48 ± 0.02	4.53	0.47 ± 0.02	4.53
*Z*-Ligustilide	99.72	98.66 ± 3.18	3.22	95.84 ± 3.73	3.89	94.19 ± 7.09	7.53
	49.86	49.75 ± 2.30	4.62	45.73 ± 2.98	6.53	45.33 ± 5.63	12.42
	0.20	0.20 ± 0.01	6.98	0.20 ± 0.01	5.86	0.18 ± 0.02	12.39
Levistilide A	133.65	130.75 ± 10.35	7.92	121.87 ± 2.34	1.92	134.59 ± 5.82	4.32
	66.82	66.05 ± 2.50	3.78	60.04 ± 2.90	4.84	59.51 ± 3.13	5.26
	0.26	0.24 ± 0.02	6.59	0.23 ± 0.01	4.83	0.24 ± 0.01	5.53
Ferulic acid	275.83	270.59 ± 11.52	4.25	273.01 ± 7.25	2.65	267.03 ± 12.54	4.69
	137.92	135.24 ± 2.82	2.08	130.44 ± 5.80	4.45	130.24 ± 5.58	4.29
	0.44	0.46 ± 0.02	3.30	0.40 ± 0.02	5.72	0.38 ± 0.02	5.72
Liquiritigenin	109.96	109.52 ± 5.78	5.28	100.24 ± 4.19	4.18	103.39 ± 4.89	4.73
	54.98	54.93 ± 2.87	5.22	52.98 ± 2.55	4.82	52.83 ± 2.92	5.53
	0.22	0.21 ± 0.02	7.80	0.21 ± 0.01	4.29	0.23 ± 0.01	3.28
Liquiritin	99.80	96.45 ± 2.60	2.69	95.60 ± 3.67	3.84	95.10 ± 3.05	3.20
	49.90	48.26 ± 3.11	6.44	47.24 ± 1.59	3.37	48.52 ± 1.78	3.67
	0.39	0.37 ± 0.02	5.06	0.40 ± 0.01	3.60	0.40 ± 0.01	3.67
Paeoniflorin	237.77	237.17 ± 6.60	2.78	231.08 ± 10.05	4.35	230.52 ± 6.66	2.89
	115.89	111.05 ± 5.21	4.74	112.57 ± 4.52	4.01	111.74 ± 4.34	3.89
	0.37	0.37 ± 0.02	5.22	0.35 ± 0.01	2.67	0.33 ± 0.01	3.00
Albiflorin	236.90	230.34 ± 9.76	4.24	234.12 ± 5.07	2.17	225.62 ± 4.52	2.00
	118.45	115.59 ± 4.47	3.87	1140.07 ± 4.96	4.35	113.38 ± 3.35	2.95
	0.37	0.37 ± 0.02	5.65	0.35 ± 0.01	3.57	0.35 ± 0.02	5.27
Oxypaeoniflorin	110.40	106.80 ± 3.52	3.30	108.06 ± 4.11	3.80	105.05 ± 2.64	2.52
	55.20	54.62 ± 1.43	2.62	52.95 ± 1.78	3.37	53.32 ± 2.50	4.68
	0.44	0.43 ± 0.02	3.61	0.42 ± 0.02	4.77	0.44 ± 0.03	6.27
Poricoic acid A	107.04	107.66 ± 3.53	3.28	107.10 ± 3.59	3.35	105.14 ± 1.60	1.52
	53.52	51.71 ± 1.40	2.72	51.89 ± 1.82	3.50	51.34 ± 1.91	3.72
	0.42	0.42 ± 0.02	4.50	0.38 ± 0.02	5.18	0.44 ± 0.02	5.57
Poricoic acid B	114.70	113.41 ± 4.31	3.80	111.42 ± 2.38	2.13	109.52 ± 3.94	3.60
	57.35	53.19 ± 4.00	7.52	53.53 ± 2.25	4.21	52.46 ± 1.27	2.43
	0.45	0.43 ± 0.03	6.80	0.42 ± 0.02	4.70	0.44 ± 0.03	7.34
Licochalcone A	107.91	107.24 ± 2.63	2.46	105.15 ± 2.91	2.77	105.15 ± 2.91	2.77
	53.96	53.79 ± 0.97	1.81	52.17 ± 3.64	6.97	50.15 ± 1.82	3.62
	0.21	0.21 ± 0.01	4.17	0.20 ± 0.01	5.35	0.21 ± 0.01	5.77

**Table 5:** Gastric administration doses of each compound.

Compound	Low Dose	Medium Dose	High Dose
	μg/kg/d	μg/kg/d	μg/kg/d
Saikosaponin A	877.72	1775.44	3550.88
Saikosaponin D	557.39	1114.78	2229.56
*Z*-Ligustilide	904.50	1809.00	3618.00
Levistilide A	62.96	125.92	251.84
Ferulic acid	28.73	57.46	114.92
Liquiritigenin	16.31	32.62	65.24
Liquiritin	163.40	326.80	653.60
Paeoniflorin	2080.62	4161.24	8322.48
Albiflorin	1994.65	3989.30	7978.60
Oxypaeoniflorin	61.78	123.56	247.12
Poricoic acid A	23.65	47.30	94.60
Poricoic acid B	11.34	22.68	45.36
Licochalcone A	5.29	10.58	21.16

**Table 6:** Mass spectrometry conditions.

Compound	Precursor Ion	Daughter Ion	Collision Voltage	RT (min)	ESI
Saikosaponin A	779.41	144.88	45	10.89	-
Saikosaponin D	779.43	439.13	65	12.36	-
*Z*-Ligustilide	190.98	76.88	40	15.75	+
Levistilide A	381.11	78.88	47	15.76	+
Ferulic acid	192.96	133.96	15	7.44	-
Liquiritigenin	254.98	90.80	27	8.78	-
Liquiritin	416.98	118.88	45	6.96	-
Paeoniflorin	479.01	120.88	20	7.87	-
Albiflorin	479.01	76.88	42	6.48	-
Oxypaeoniflorin	495.05	136.88	29	5.17	-
Poricoic acid A	497.30	211.05	43	14.83	-
Poricoic acid B	483.31	409.13	30	14.27	-
Licochalcone A	339.03	92.88	44	12.47	+

## Data Availability

The data presented in this study are available on request from the corresponding author.

## Supplementary Materials

The following supporting information can be downloaded at: https://www.mdpi.com/article/10.3390/molecules29061230/s1. Figure S1. Exclusive chromatogram of components to be tested (A: chromatogram of QC sample, B: chromatogram of sample, C: chromatogram of blank plasma); Table S1. Main pharmacokinetic parameters of the control groups after administration of JYP; Table S2. Main pharmacokinetic parameters of ADHD model group after administration of JYP.

## Abbreviations

JYPs: Jieyu Pills, CM: Chinese Medicine, ESI: electrospray ionization, UPLC-MS: Ultra-high-performance liquid chromatography–mass spectrometry, SD: Sprague Dawley, ADHD: attention deficit hyperactivity disorder, TIC: total ion current, QC: quality control, AUC: area under the plasma concentration–time curve, MRT: mean residence time. C_max_: maximum concentration, t_1/2_: half-life.
