# Supplementary material for: Comparative Pharmacokinetics Research of 13 Bioactive Components of Jieyu Pills in Control and Attention Deficit Hyperactivity Disorder Model Rats Based on UPLC-Orbitrap Fusion MS
Source: Molecules. 2024 Mar 10;29(6):1230. doi: 10.3390/molecules29061230 (PMC10976160; doi:10.3390/molecules29061230)
Supplement: Supplementary file 1 [file molecules-29-01230-s001.zip › molecules-2865582-supplementary.pdf]

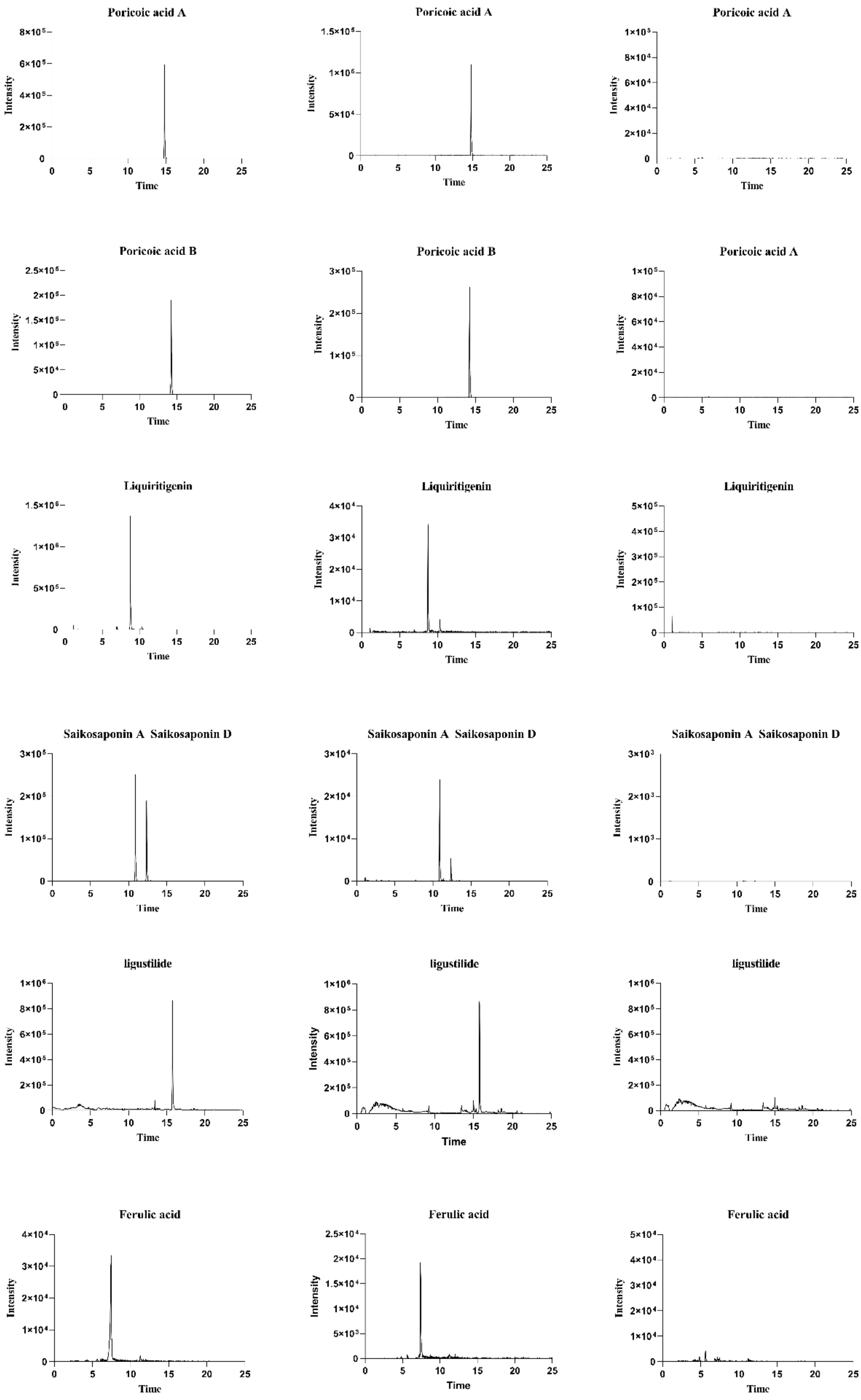
Poricoic acid A
Intensity
Time
8×10⁵
6×10⁵
4×10⁵
2×10⁵
0
5
10
15
20
25
Poricoic acid A
1.5×10⁶
1×10⁶
5×10⁴
Poricoic acid A
1×10⁵
8×10⁴
6×10⁴
4×10⁴
2×10⁴
Poricoic acid B
2.5×10⁶
2×10⁵
1.5×10⁵
1×10⁵
5×10⁴
Poricoic acid B
3×10⁵
2×10⁵
1×10⁵
Poricoic acid A
Liquiritigenin
1.5×10⁶
1×10⁶
5×10⁵
Liquiritigenin
4×10⁴
3×10⁴
2×10⁴
1×10⁴
Liquiritigenin
5×10⁵
4×10⁵
3×10⁵
2×10⁵
1×10⁵
Saikosaponin A Saikosaponin D
3×10⁵
2×10⁵
1×10⁵
Saikosaponin A Saikosaponin D
3×10⁴
2×10⁴
1×10⁴
Saikosaponin A Saikosaponin D
3×10³
2×10³
1×10³
ligustilide
1×10⁶
8×10⁵
6×10⁵
4×10⁵
2×10⁵
ligustilide
ligustilide
Ferulic acid
4×10⁴
3×10⁴
2×10⁴
1×10⁴
Ferulic acid
2.5×10⁴
2×10⁴
1.5×10⁴
1×10⁴
5×10³
Ferulic acid
5×10⁴
4×10⁴
3×10⁴
2×10⁴
1×10⁴

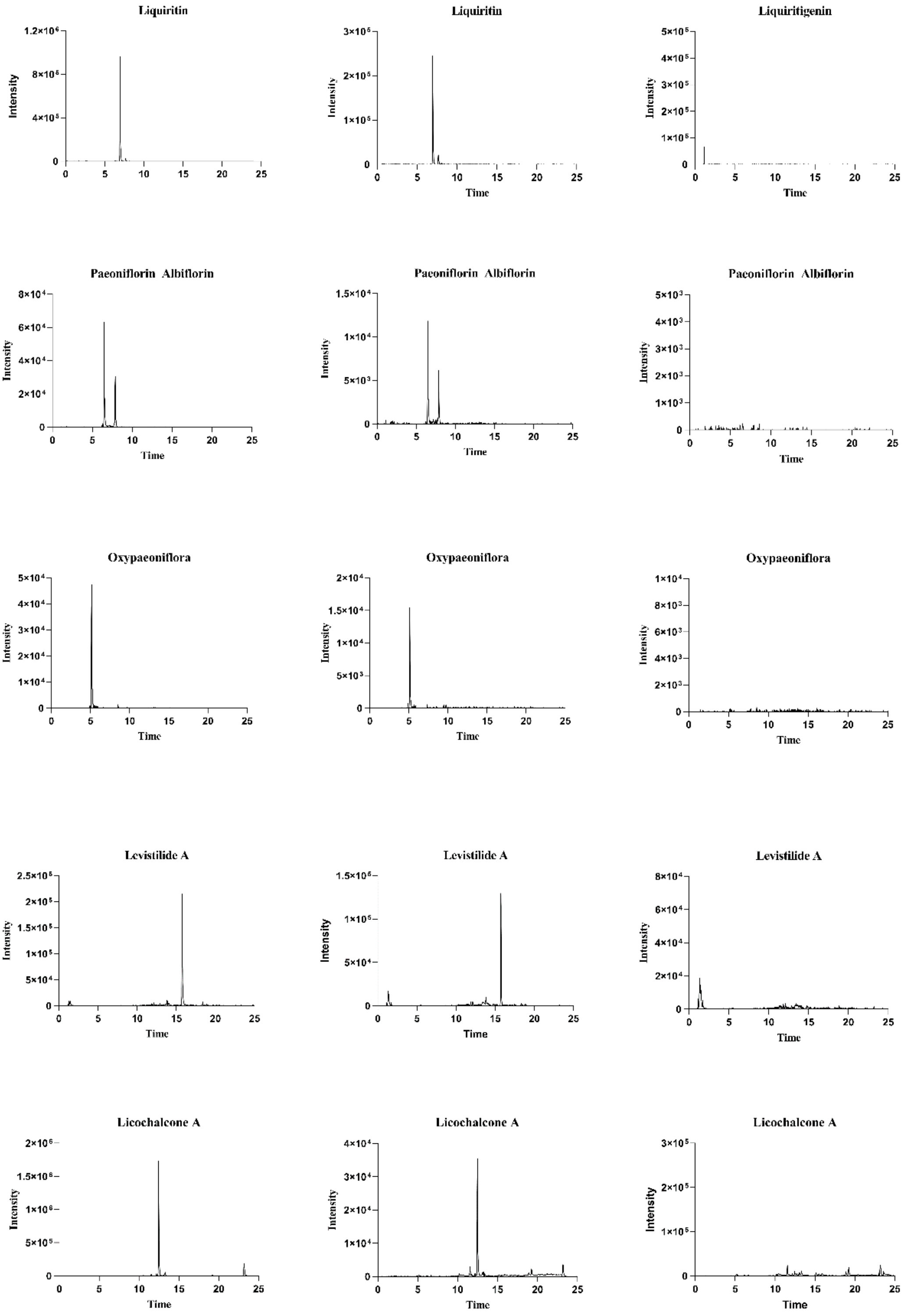

A B C

Figure S1. Exclusive chromatogram of components to be tested (A: chromatogram of QC sample, B: chromatogram of sample, C: chromatogram of blank plasma)

Table S1. Main pharmacokinetic parameters of the control groups after administration of JYP.

Compounds	C_{max} (ng/mL)	$AUC_{(0-t)}$ (ng/mL·h)	$AUC_{(0-\infty)}$ (ng/mL·h)	T_{max} (h)	$t_{1/2}$ (h)	Clearances (mL / h)	$MRT_{(0-t)}$ (h)	$MRT_{(0-\infty)}$ (h)	Vss (mL)	*$AUMC_{(0-t)}$* ng/mL*(h)²	*$AUMC_{(0-\alpha)}$* ng/mL*(h)²
**Saikosaponin A**	38.91±10.43<br>47.41±11.72<br>67.38±14.24	119.62±40.57<br>148.78±49.46<br>220.57±42.46	134.56±49.83<br>177.04±46.52<br>244.87±44.68	0.53±0.22<br>0.39±0.17<br>0.54±0.10	7.52±2.66<br>10.82±9.21<br>7.80±3.30	2262.98±1068.56<br>3201.17±894.28<br>4464.90±760.17	6.55±1.64<br>6.42±0.60<br>6.07±1.19	9.57±3.88<br>13.26±9.41<br>9.11±3.69	21125.87±12315.99<br>42581.43±30523.07<br>39888.83±14247.9z	792.28±390.55<br>935.87±261.97<br>1333.05±317.31	1355.37±1006.84<br>2307.92±1659.75<br>2265.45±1085.76
**Saikosaponin D**	21.53±5.52<br>30.14±6.87<br>41.77±17.69	79.57±44.57<br>96.80±29.29<br>115.72±12.30	97.26±56.51<br>116.99±33.20<br>156.97±34.87	0.43±0.23<br>0.54±0.10<br>0.33±0.13	11.43±4.14<br>10.86±6.69<br>13.70±5.61	2081.51±796.95<br>3109.21±1089.09<br>4477.49±1178.04	7.08±1.57<br>6.87±0.65<br>7.10±1.12	13.36±4.81<br>13.34±6.43<br>16.61±6.52	27762.67±16738.07<br>41378.73±24017.48<br>68432.42±15413.77	592.40±473.29<br>662.12±211.50<br>830.12±197.07	1331.60±1006.07<br>1586.66±988.85<br>2785.93±1472.47
**Z-Ligustilide**	3.37±1.11<br>4.47±1.90<br>5.27±1.27	28.73±8.22<br>32.70±6.04<br>36.53±7.42	40.81±9.33<br>58.61±20.74<br>53.40±9.38	1.67±0.52<br>1.17±0.41<br>1.13±0.44	15.40±5.99<br>23.99±16.94<br>16.44±5.40	9559.18±2479.10<br>10223.65±3357.69<br>20983.77±4562.19	8.54±1.12<br>8.96±0.63<br>8.76±0.37	20.38±8.24<br>32.03±20.88<br>21.57±6.20	190594.33±82167.70<br>280622.83±89334.71<br>448255.00±139953.43	244.45±72.75<br>291.74±50.18<br>319.41±64.35	839.61±369.47<br>2206.47±2197.92<br>1163.41±438.24
**Levistilide A**	1.79±0.66<br>2.86±0.52<br>4.60±2.01	12.91±4.96<br>21.47±2.97<br>36.70±11.31	25.25±7.16<br>34.23±5.08<br>59.83±23.13	1.17±0.41<br>0.92±0.13<br>1.67±0.52	14.36±4.36<br>16.45±7.30<br>22.15±26.88	794.02±199.77<br>1123.37±161.30<br>1455.57±663.81	9.51±1.01<br>9.23±0.46<br>8.92±1.16	22.87±7.04<br>24.13±8.96<br>31.23±38.06	17878.07±6212.70<br>26341.48±7666.95<br>33463.62±24837.03	150.01±45.26<br>197.79±26.58<br>323.93±97.01	584.44±254.39<br>849.62±407.65<br>2493.49±4004.18

**Ferulic acid**	26.13±9.31<br>33.03±6.66<br>41.33±6.94	112.20±29.20<br>164.81±27.71<br>153.34±31.07	167.65±82.40<br>207.47±62.03<br>218.79±56.06	0.11±0.07<br>0.15±0.03<br>0.33±0.13	15.83±15.51<br>9.53±4.55<br>15.42±5.57	60.71±24.99<br>88.60±22.65<br>167.21±46.46	8.20±0.89<br>7.97±0.79<br>7.78±1.05	21.77±21.42<br>13.84±5.77<br>19.29±4.66	1020.61±427.20<br>1137.02±170.35<br>3090.77±631.45	920.92±270.38<br>1316.71±312.22<br>1199.51±316.16	4989.84±7833.58<br>3143.66±2448.63<br>4375.94±1872.57
**Liquiritigenin**	2.95±1.02<br>4.39±0.47<br>5.27±2.18	24.28±7.57<br>30.41±5.25<br>33.94±6.26	37.90±10.24<br>50.33±15.38<br>48.45±10.22	0.58±0.13<br>0.63±0.21<br>0.58±0.13	16.81±4.56<br>19.40±15.30<br>13.44±3.91	139.00±44.74<br>206.08±46.72<br>416.54±72.82	9.56±0.40<br>9.27±0.40<br>9.08±1.02	24.13±6.47<br>27.60±20.69<br>19.47±5.47	3476.06±2016.81<br>4980.58±1943.61<br>7947.55±2008.46	231.13±70.38<br>280.27±38.86<br>305.66±51.85	892.16±251.11<br>1640.81±1923.68<br>969.22±453.25
**Liquiritin**	4.21±1.22<br>6.35±1.64<br>7.83±3.33	28.27±5.08<br>30.83±8.16<br>40.61±13.09	37.25±7.36<br>44.11±11.65<br>56.01±12.24	0.49±0.19<br>0.38±0.14<br>0.42±0.13	10.95±2.30<br>11.12±2.84<br>13.90±3.40	1366.04±306.58<br>2363.69±690.44<br>3640.48±791.51	8.60±0.60<br>7.09±1.11<br>8.96±1.02	16.05±3.72<br>16.55±5.41<br>19.32±5.01	21368.90±3823.75<br>32211.83±13820.62<br>72786.07±31673.07	242.78±42.16<br>278.48±81.31<br>355.04±86.72	609.90±217.99<br>764.39±381.98<br>1043.82±188.119
**Paeoniflorin**	20.87±5.05<br>28.02±12.40<br>50.87±10.98	77.44±19.10<br>104.91±34.22<br>179.49±32.14	92.68±25.59<br>121.52±40.73<br>202.71±47.28	0.71±0.10<br>0.67±0.13<br>0.79±0.10	10.38±5.59<br>9.96±2.44<br>7.93±2.12	7146.41±1816.51<br>10962.90±2498.31<br>12850.86±2810.92	6.69±.1.11<br>6.45±0.78<br>6.42±1.11	12.13±3.83<br>10.82±1.69<br>9.59±3.21	84257.15±26998.98<br>118512.68±32910.62<br>119548.08±32481.96	515.74±155.66<br>657.24±121.76<br>1155.62±333.44	1159.98±582.94<br>1315.91±477.44<br>2026.57±1173.91
**Albiflorin**	29.10±12.43<br>31.97±7.33<br>41.76±7.73	113.28±34.45<br>136.03±51.60<br>195.87±30.38	145.97±34.53<br>179.64±67.31<br>259.43±66.27	0.71±0.10<br>0.71±0.10<br>1.08±0.47	12.20±8.94<br>13.32±8.18<br>12.74±7.86	4310.26±1096.22<br>7593.60±3243.27<br>9666.39±2118.21	7.49±1.37<br>7.66±0.75<br>7.19±0.47	16.35±10.03<br>17.63±8.10<br>16.23±7.55	67797.93±37215.77<br>149394.60±144699.53<br>147016.78±46808.40	850.80±299.27<br>1063.52±483.38<br>1414.64±276.30	2439.47±1660.72<br>2994.60±1496.88<br>4549.04±3389.12

**Oxypaeoniflorin**	11.00±5.55	43.15±6.64	56.34±16.77	0.63±0.21	11.63±7.54	347.97±78.14	7.43±1.20	15.41±7.45	4942.84±1353.80	317.45±50.32	960.89±804.49
	13.77±4.74	58.53±13.77	71.14±17.02	0.54±0.10	9.23±2.09	543.42±114.7	7.55±0.90	12.86±2.29	6979.24±1997.60	444.62±135.55	919.38±307.57
	21.56±4.62	87.76±11.69	116.14±5.19	0.50±0.16	13.51±5.15	639.43±29.38	7.88±0.35	17.12±5.17	10992.03±3569.08	690.02±87.45	1980.07±571.31
**Poricoic acid A**	7.81±4.17	58.34±32.40	69.77±35.24	2.67±1.51	8.56±2.26	128.09±61.58	7.75±0.56	1521.44±665.04	443.98±219.49	443.98±219.49	794.53±355.07
	15.42±4.34	72.48±16.40	82.77±20.26	1.04±0.51	7.61±2.67	180.08±44.30	6.61±0.81	1783.08±521.01	481.57±135.07	481.57±135.07	860.09±370.64
	22.57±4.45	99.97±21.03	112.84±26.44	1.17±0.65	7.95±2.00	268.18±87.45	6.79±0.52	2589.92±548.64	682.54±172.01	682.54±172.01	1154.08±438.70
**Poricoic acid B**	13.92±3.90	75.66±26.78	87.37±18.47	2.00±1.10	12.23±13.25	40.53±9.10	7.03±0.42	15.07±13.62	679.29±748.52	529.52±188.12	1182.43±817.51
	28.41±8.52	119.95±33.34	133.19±32.48	1.67±1.21	9.63±3.01	53.51±12.19	5.74±0.86	9.23±1.94	506.22±195.93	679.25±172.30	1195.18±213.22
	35.95±8.74	141.91±41.47	159.33±46.64	1.17±0.41	10.75±6.42	91.65±27.11	5.67±0.98	9.63±1.90	869.19±245.83	810.63±320.58	1551.8±565.53
**Licochalcone A**	1.08±0.20	11.43±1.69	17.55±2.70	2.17±0.98	13.43±1.54	92.37±15.28	9.44±0.40	21.28±2.11	1954.78±297.39	108.18±18.41	375.00±71.45
	1.47±0.11	16.35±1.88	28.44±7.01	1.83±0.41	19.54±6.07	116.55±24.65	9.63±0.13	27.60±7.20	3084.37±231.29	157.42±18.66	825.38±455.76
	2.94±0.97	25.91±9.50	37.37±17.13	3.33±1.03	14.11±3.03	194.20±66.59	9.03±0.59	19.42±3.88	3631.94±1163.37	237.25±103.25	767.46±504.79

Table S2. Main pharmacokinetic parameters of ADHD model group after administration of JYP.

Compounds	C_{max} (ng/mL)	$AUC_{(0-t)}$ (ng/mL·h)	$AUC_{(0-\infty)}$ (ng/mL·h)	T_{max} (h)	$t_{1/2}$ (h)	Clearances (mL / h)	$MRT_{(0-t)}$ (h)	$MRT_{(0-\infty)}$ (h)	Vss (mL)	*$AUMC_{(0-t)}$* ng/mL*(h)^2	*$AUMC_{(0-\infty)}$* ng/mL*(h)^2
**Saikosaponin A**	40.88±22.88<br>52.96±19.17<br>73.09±22.62	163.91±57.46<br>240.37±96.61<br>307.06±55.58*	232.86±91.18<br>324.31±131.07*<br>387.10±78.69**	0.50±0.16<br>0.79±0.19**<br>0.58±0.13	13.28±7.06<br>12.24±2.82<br>10.30±4.24	1291.22±525.61<br>2143.07±1609.00<br>2855.71±621.15**	8.75±1.60*<br>8.13±1.23*<br>7.95±1.20*	19.45±9.93<br>16.82±3.55<br>14.51±5.42	23782.60±13625.69<br>35282.72±25449.21<br>40360.07±12917.92	1392.40±460.59*<br>1937.54±771.89*<br>2395.92±288.80**	4853.64±3957.39<br>5558.75±2961.42*<br>5783.46±3073.92*
**Saikosaponin D**	22.57±2.41<br>33.11±3.36<br>41.49±12.39	91.33±24.62<br>145.21±32.82*<br>156.01±38.62*	132.27±32.26<br>187.52±55.24*<br>200.87±50.03	0.58±0.13<br>0.54±0.25<br>0.38±0.14	14.99±5.90<br>10.61±4.96<br>10.31±2.55	1343.52±399.07<br>1952.91±715.40<br>3506.08±856.33	8.43±0.96<br>7.80±1.36<br>8.26±0.54*	20.27±5.60*<br>14.80±6.52<br>15.15±2.75	26784.08±8767.24<br>26532.70±7619.14<br>53202.65±17657.23	761.68±201.22<br>1149.72±377.93*<br>1294.18±361.60*	2701.81±992.13*<br>3004.18±2085.30<br>3035.93±850.28
**Z-Ligustilide**	4.07±1.85<br>5.57±2.26<br>6.63±1.78	36.70±18.17<br>44.73±18.17<br>49.53±16.75	49.28±14.69<br>62.20±24.53<br>69.65±27.09	1.67±1.21<br>1.13±.44<br>0.96±0.10	12.78±3.20<br>14.63±7.18<br>12.48±3.72	8062.61±2184.22<br>10040.73±4395.33<br>19177.75±11691.01	8.90±1.12<br>8.72±0.80<br>8.90±1.50	17.86±4.80<br>19.97±7.80<br>17.99±5.00	145289.80±58639.01<br>207173.33±141485.31<br>306975.33±95317.08	322.64±100.23<br>383.83±150.84<br>458.69±201.28	870.35±311.46<br>1224.58±657.13<br>1348.99±756.73

**Levistilide A**	2.61±0.78<br>3.46±1.33<br>5.48±1.21	23.05±7.24<br>22.07±7.91<br>34.83±3.46	43.19±21.11<br>42.96±11.90<br>42.60±3.94	1.04±0.51<br>1.13±0.44<br>0.88±0.14**	21.83±11.87<br>30.15±23.34<br>9.51±2.70	583.82±391.47<br>940.83±272.59<br>1786.84±173.43	9.48±1.38<br>9.25±0.28<br>8.15±0.93	30.29±15.78<br>41.03±31.64<br>13.65±3.32	13560.55±3696.98<br>35525.00±20523.09<br>24223.78±5474.48	221.98±85.74<br>202.94±68.72<br>282.51±29.20	1547.01±1200.74<br>1908.62±1879.83<br>585.23±168.30
**Ferulic acid**	28.60±10.23<br>37.61±7.18<br>40.17±7.44	174.27±35.55**<br>138.57±45.63<br>207.00±49.04*	240.77±94.77<br>176.71±49.42<br>270.69±96.33	1.94±2.47<br>0.26±0.13<br>0.57±0.71	10.77±5.14**<br>8.30±1.95<br>8.91±3.85*	41.09±16.96<br>106.16±38.60<br>142.94±57.33	8.41±2.19<br>8.47±1.41<br>8.41±1.18	16.16±8.17<br>14.43±5.48<br>14.73±5.85	554.79±128.74*<br>1504.68±634.79<br>1957.50±726.62*	1517.48±592.99*<br>1141.49±272.08<br>1721.27±433.91*	4511.05±3598.15<br>2579.37±1378.12<br>4310.54±3003.20
**Liquiritigenin**	1.80±0.72*<br>2.77±0.62**<br>3.78±0.55	17.22±5.10<br>19.69±2.73*<br>21.95±4.95**	29.56±8.94<br>29.15±6.87<br>36.98±11.35	0.71±0.19<br>0.50±0.16<br>0.58±0.13	17.74±5.19<br>14.38±6.03<br>18.87±10.81	178.52±55.32<br>349.45±70.98**<br>562.94±135.21*	9.97±1.24<br>9.08±0.96<br>9.34±0.93	25.79±7.03<br>20.75±7.90<br>27.05±15.24	3869.35±2209.32<br>6883.75±1548.30<br>13934.89±4664.02*	169.83±48.23<br>178.55±30.04<br>201.96±28.84**	776.47±351.07<br>642.59±413.59<br>1121.62±1058.33
**Liquiritin**	2.51±1.07*<br>3.66±0.64**<br>7.17±3.53	14.40±3.71**<br>15.36±2.24**<br>20.39±4.01**	29.45±16.14<br>22.76±3.51**<br>27.75±6.02**	0.53±0.27<br>0.54±0.25<br>0.51±0.73	22.75±18.27<br>13.49±4.39<br>11.71±3.79	1949.37±695.17<br>4398.06±748.59**<br>7361.22±1682.00**	9.96±1.77<br>8.80±0.95<br>8.19±0.87	31.67±22.77<br>20.79±5.25<br>16.93±4.17	34996.20±28160.02<br>76300.87±39923.09*<br>121685.50±27070.54*	139.02±20.98**<br>134.13±15.77**<br>166.05±32.15**	417.87±153.13<br>478.30±156.32<br>479.91±186.34**

**Paeoniflorin**	39.57±5.34<br>44.88±9.70*<br>63.49±22.74*	144.54±57.20<br>173.70±75.99<br>204.42±59.92	226.29±191.54<br>234.62±126.43<br>231.05±63.54	0.63±0.31<br>0.46±0.33<br>0.29±0.10**	14.25±10.91<br>9.82±5.35<br>7.21±2.51	3673.59±1648.88<br>7279.35±5603.80<br>11012.36±2893.69	7.61±1.59<br>6.95±1.67<br>6.51±0.51*	18.77±14.99<br>13.71±8.05<br>9.89±2.27	53940.15±25027.14<br>84884.42±41057.56<br>109397.02±35687.33	1173.15±749.63<br>1245.17±723.09<br>1309.93±289.55	6541.21±11431.06<br>3775.73±3756.09<br>2274.30±887.26
**Albiflorin**	39.57±5.34<br>44.88±9.70*<br>63.49±22.74*	144.54±57.20<br>173.70±75.99<br>204.42±59.92	226.29±191.54<br>234.62±126.43<br>231.05±63.54	0.63±0.31<br>0.46±0.33<br>0.29±0.10**	14.25±10.91<br>9.82±5.35<br>7.21±2.51	3673.59±1648.88<br>7279.35±5603.80<br>11012.36±2893.69	7.61±1.59<br>6.95±1.67<br>6.51±0.51*	18.77±14.99<br>13.71±8.05<br>9.89±2.27	53940.15±25027.14<br>84884.42±41057.56<br>109397.02±35687.33	1173.15±749.63<br>1245.17±723.09<br>1309.93±289.55	6541.21±11431.06<br>3775.73±3756.09<br>2274.30±887.26
**Oxypaeoniflorin**	8.53±4.38<br>12.69±7.45<br>25.73±9.19	47.39±13.88<br>81.72±35.35<br>112.83±43.59	53.84±11.65<br>96.39±28.89<br>127.95±45.72	2.71±2.60<br>1.83±1.17*<br>1.08±0.49*	9.66±8.61<br>12.77±12.31<br>8.11±3.60	360.46±91.80<br>414.32±120.17<br>671.36±310.16	7.57±1.97<br>7.14±1.32<br>7.10±1.14	12.55±5.31<br>15.81±14.32<br>10.91±3.15*	4725.76±2732.40<br>7178.93±7592.04<br>7494.03±4533.51	363.05±150.34<br>559.87±206.42<br>786.02±296.19	647.25±230.17<br>1363.45±990.24<br>1367.87±579.03
**Poricoic acid A**	15.53±4.05**<br>26.24±6.09**<br>30.54±16.03	89.10±35.55<br>100.92±47.28<br>194.98±85.11*	99.84±41.52<br>152.53±89.34<br>225.13±98.33*	0.71±0.37*<br>1.71±2.11<br>4.42±2.50*	6.99±1.97<br>10.32±8.55<br>6.86±3.37	84.44±42.36<br>117.58±56.05<br>178.73±160.12	7.86±1.25<br>7.52±1.73<br>8.05±1.46	10.75±2.43<br>14.33±11.30<br>11.38±4.62	925.63±607.13<br>2518.64±3044.51<br>1826.27±1334.95	705.71±328.26<br>788.38±501.00<br>1567.88±729.78	1082.34±582.13<br>2959.99±4043.39<br>2703.46±1701.67
**Poricoic acid B**	17.37±8.26<br>28.04±7.19<br>38.23±13.61	110.35±56.12<br>141.06±44.89<br>241.88±68.85*	136.15±93.67<br>153.72±47.26<br>250.00±69.23*	0.75±0.39*<br>2.67±2.58<br>5±1.10**	8.04±4.49<br>5.98±2.46*<br>4.23±1.12*	34.42±18.58<br>48.05±15.22<br>58.10±16.47*	8.56±1.68<br>7.20±2.12<br>7.04±0.43*	12.63±6.43<br>9.38±3.50<br>7.84±0.93	394.18±220.52<br>427.28±134.62<br>459.11±151.78**	948.35±595.70<br>1064.19±564.04<br>1696.05±470.24*	2103.24±2792.61<br>1509.97±839.70<br>1946.42±524.46

**Licochalcone A**	1.45±0.20*	14.85±3.04*	28.80±6.93**	1.08±0.47*	23.98±10.62*	58.07±15.14**	10.25±0.51*	34.36±13.80*	1891.10±569.09	151.85±28.97*	1038.56±608.98*
	2.50±0.53**	18.32±4.23	25.84±6.29	2.17±0.98	13.48±7.30	129.92±35.81	8.41±0.75**	19.09±7.67	2365.98±778.28	155.16±44.11	512.63±272.88
	3.45±0.70	23.09±5.76	30.73±5.56	2.67±1.03	12.90±4.17	211.57±33.51	8.41±0.95	17.35±4.38	3697.79±1176.34	195.07±60.32	527.62±128.84
